# US Power Production at Risk from Water Stress in a Changing Climate

**DOI:** 10.1038/s41598-017-12133-9

**Published:** 2017-09-20

**Authors:** Poulomi Ganguli, Devashish Kumar, Auroop R. Ganguly

**Affiliations:** 1 Sustainability and Data Sciences Laboratory (SDS Lab), Department of Civil and Environmental Engineering, Northeastern University, Boston, 02115 Massachusetts United States; 2 Present Address: GFZ German Research Centre for Geosciences, Section 5.4 Hydrology, 14473 Potsdam, Germany

## Abstract

Thermoelectric power production in the United States primarily relies on wet-cooled plants, which in turn require water below prescribed design temperatures, both for cooling and operational efficiency. Thus, power production in US remains particularly vulnerable to water scarcity and rising stream temperatures under climate change and variability. Previous studies on the climate-water-energy nexus have primarily focused on mid- to end-century horizons and have not considered the full range of uncertainty in climate projections. Technology managers and energy policy makers are increasingly interested in the decadal time scales to understand adaptation challenges and investment strategies. Here we develop a new approach that relies on a novel multivariate water stress index, which considers the joint probability of warmer and scarcer water, and computes uncertainties arising from climate model imperfections and intrinsic variability. Our assessments over contiguous US suggest consistent increase in water stress for power production with about 27% of the production severely impacted by 2030s.

## Introduction

Presently, 91% (3500 million MW h)^1^ of total electricity in the United States is generated by fossil-fueled thermoelectric power plants, which use 45% (~161 billion gallons per day)^2^ of total freshwater withdrawal (the single largest use of fresh water), 90% of which is used for cooling. High temperature of intake water directly affects the energy conversion efficiency^3^. Environmental regulations^4^ put limits on the temperature of discharged water to protect aquatic lives. The large amount of water required^1^ at low temperatures highlights the importance of abundant water resources for uninterrupted operation of a power plant^5^. During the periods of low flows, the plants are forced to operate at a reduced capacity^1,5,6^ and have to be shut down temporarily if water temperature exceeds the certain operational threshold set by the U.S. EPA (Environment Protection Agency of the US)^7–9^. The demands for industrial energy consumption is projected to increase by more than 25% by 2040^10^; this raises concern how much freshwater^11–13^ would be available for thermoelectric power generation. In fact, limited freshwater supplies are one of the constraints for installation of new thermoelectric facilities in certain regions of the US^14,15^. About thirty-five coal- and nuclear-fueled power plants were shut down completely or had to curtail power production because of water problems from 2006–2013^16^.

Droughts-induced^17,18^ water scarcity and heat-waves driven warm water have already impacted power productions in the several parts of US, including Texas^18,19^ and California^20^. Future thermoelectric power production will depend on the availability of sufficient water resources, which will directly be impacted under climate change^6,13,21–27^. Changes in precipitation patterns along with increased evapotranspiration rates^28^ have already reduced freshwater availability in the certain regions of US. Freshwater availability^11^ and scarcity^29^, and water temperature^30^ under climate change has been studied in the context of food security^23,31^, droughts^32^, and power production^13,22,24,25^. Using hydrological (Variable Infiltration Capacity [VIC]) and one-dimensional stream temperature^33^ models forced with outputs from global climate models (GCMs), Vliet *et al*.^24^ showed the vulnerability of thermoelectric power production in the US and Europe for mid- and end-of-the century time horizons. Using one and three-dimensional hydrodynamic models (SIMSTRAT and Delft3D-Flow), Love Råman Vinnå *et al*.^34^ found a sharp increase in seasonal temperature, locally up to 3.4 °C and system-wide ~0.3 °C on the medium sized lake (Lake Biel) in Switzerland, influenced by an upstream nuclear power plant. Bartos and Chester^25^ employed the Vliet *et al*.^24^ modeling framework to study the impacts of climate change on power supply from five energy generation technologies in the western US. Blanc *et al*.^26^ employed extended Integrated Global System model to map US water resources and identified water-stressed regions based on water requirement driven by population and economic growth.

Previous studies^7,25,30^ used climate data from only a few selected GCMs and did not explore the full range of multi-model response variability, which represents limitations of our understanding and modeling of physical processes in the climate models. Hydrological modeling of river flows and stream temperatures^33^ at daily scale introduces additional uncertainty in their projections. Furthermore, these studies^24,25^ were focused on mid-to-end-of-the-century time horizons; insights derived at long-term may not be credible to develop adaptation strategies for near-term (0–30 years)^35,36^ planning horizons. Besides, the combined effects of water stressors on future power production were not considered. Climate internal variability^37^, which contributes significantly to uncertainty in projections of precipitation^38,39^ especially at regional scales, was also not considered. The Integrated Assessment Models (IAMs)^27,40^ combine the drivers of water supply and water demand in a single unified framework; however, the inability of IAMs to simulate climate extremes may preclude their utility in mapping water stressed regions.

Earlier studies on the effects of water stressors on thermal power production either considered water scarcity^11–13,41^ or rising stream temperature^8,9,34^ alone. Each of the water stressors alone can severely impact power production; nevertheless, the co-occurrence of scarcer and warmer water is expected to amplify the risks and hence, increase the vulnerability of thermoelectric power plants. This study fills the gap in the literature. Here we study the concurrent effects of scarcer and warmer water to assess the vulnerability of thermoelectric power plants using a new dimensionless multivariate water stress index (see Methods). The index is motivated from the multivariate^42–45^ characterization of droughts^46,47^ and has been for the first time applied in the context of power production.

## Results

Here we estimate freshwater availability based on relatively simple hydrologic mass balance as opposed to the more heavily-parameterized physically-based hydrological models^7,25,30,34^. Our implicit hypothesis is that while the dominant effects may be captured in a more generalizable manner with relatively simple calculations, parameter misspecifications could be a concern for the more complex models. Future studies may need to examine the hypothesis more comprehensively, ideally with a suite of hydrologic models and approaches of disparate complexities. Our relatively simple method utilizes outputs from GCMs to obtain a first-order estimate of freshwater availability at regional scales (at 2-degree spatial resolution). The inherent assumption underlying the choice between a relatively simple versus more complex models or approaches in this context may be viewed as an example of the well-known bias-variance tradeoff. The more precise (hydrological models) are expected to have less bias (especially if they can capture key processes) but more variance (owing to added complexity and possible model misspecification), when compared to less variance but possibly higher bias in a simpler (mass balance type) estimate. Thus, a complex model may not be able to generalize well, especially in situations where both our process understanding and the available data are incomplete at best. Our rationale for regional mass balance is derived from the assumption that if a power plant is affected because of local water scarcity, water can be transported from remote sources. An added advantage of computing freshwater availability directly from GCMs is that it preserves the fully-coupled interaction between atmosphere, ocean, land, and sea-ice; which is why the approach has been advocated in certain previous studies^11,12,41,48,49^. In this study, water demand refers to fresh water availability for power production, and water consumption indicates any portion of water that is not returned to the original water source after being withdrawn; water consumption is not explicitly considered here.

### Regional variation in water scarcity and stream temperature across the contiguous United States

**Figure 1:**
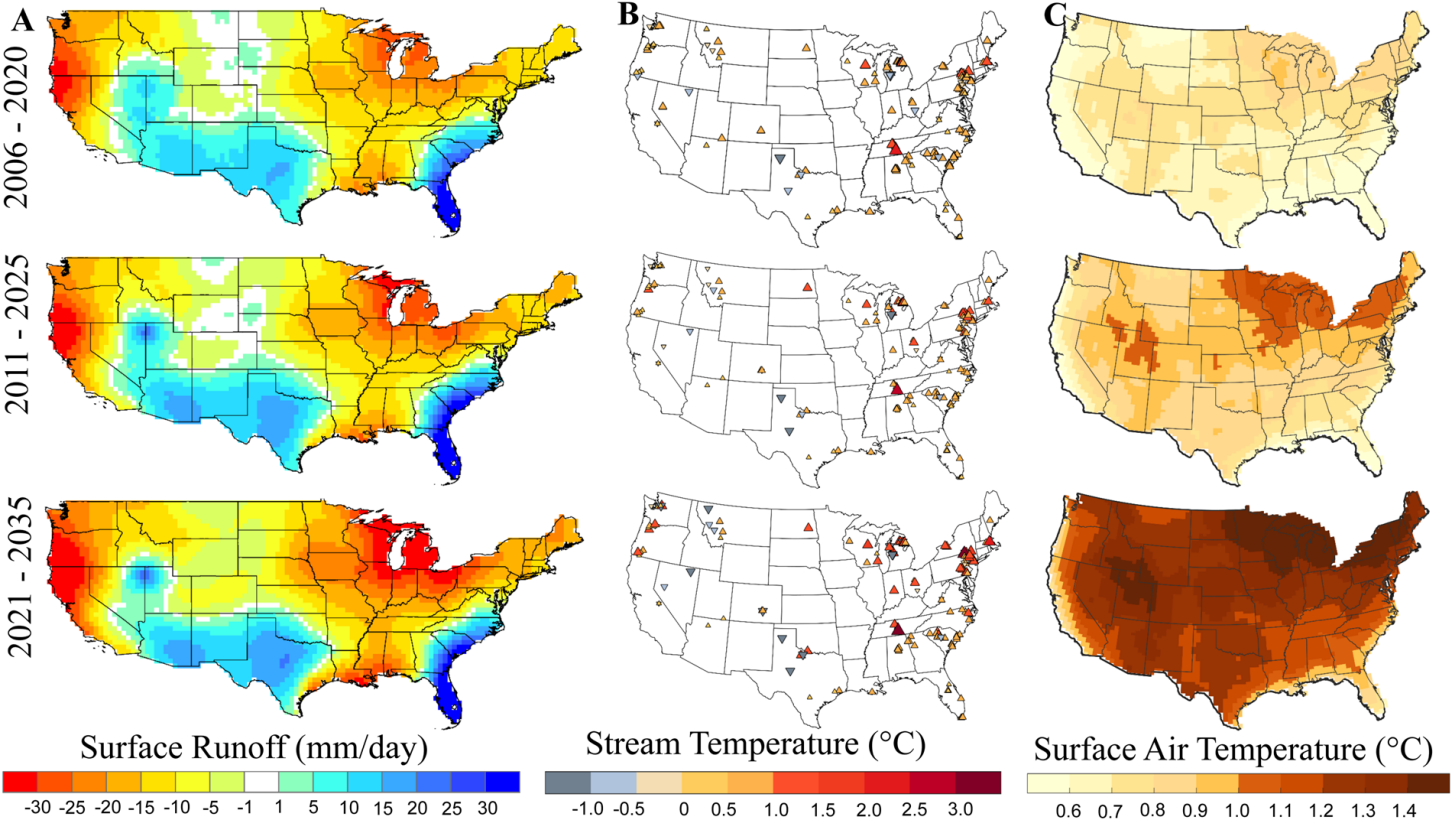
Spatial profile of water scarcity and stream temperature over near-term projection horizons. **(A)** Projected changes in low surface runoff (10^th^ percentile of all climate realizations [see Methods]) during 2006–2020 (top), 2011–2025 (middle), and 2021–2035 (bottom), relative to current estimates (1991–2005). Calculations are performed in MATLAB 2015a (Version 8.5, http://www.mathworks.com) [Software]. Shades of blue show positive changes in future freshwater availability relative to current estimates, but they do not necessarily indicate water surplus. **(B)** Same as in (**A**) but for projected changes in high stream temperature (90^th^ percentile of all climate simulations). The red (blue)-colored upward (downward) triangles in (**B**) indicate increase (decrease) in stream temperature. (**C**) Same as in **(A**) but for projected changes in 2-meter surface air temperature (90^th^ percentile). Spatial patterns of current estimates are shown in Figures S1, S2 and S4. Maps are generated using MATLAB 2015a (Version 8.5, http://www.mathworks.com) and ArcGIS Desktop (Version 10.3.1, http://www.esri.com). Finally, all these maps are organized and labelled in Adobe Photoshop CS Desktop (Version 5.1, https://www.adobe.com) [Software].

The projected changes in low surface runoff and high stream temperature show drying and warming patterns over most regions (Fig. 1) over the next three decades (2006–2035). Surface runoff or freshwater is estimated^11–13,41,49^ as the difference between monthly precipitation and evapotranspiration, in which precipitation exceeds evapotranspiration. The current estimates of freshwater are obtained from *historical* ^50^ climate simulations, and future estimates are obtained from climate projections generated using four *representative concentration pathways* (RCPs-2.6, 4.5, 6, and 8.5)^50,51^ (Table S1). Here we characterize low flows as 10^th^ percentile^7,29,47,52,53^ of surface runoff estimated using all climate realizations, and high stream temperature as 90^th^ percentile of monthly mean stream temperature time series. Future stream temperatures are predicted using Support Vector Regression (SVR) model (see Methods); the functional relationship for SVR model is developed between observed stream temperatures at the United States Geological Survey (USGS) gauge stations and historical 2-meter surface air temperatures^54,55^ from climate models (Table S2).

A snapshot of current climatology (1991–2005) of low surface runoff (Figure S1a) shows varying degree of water scarcity over most regions. Severe drying patterns are observed over parts of the South, Southeast, West, Southwest, and Central^56^; coincidentally, most of the thermoelectric power plants are also concentrated in these regions. Not surprisingly, about 35 power plants were either shut down or had to curtail their production due to water problems from 2006–2013. To put the results in perspective, we compare low flows (10^th^ percentile) with median estimates (Figure S1b; 50^th^ percentile), which show water surplus over several regions except parts of Florida, California, and Texas. The relative changes in low flows in the future (Fig. 1A; 2006–2035) compared to current estimates (Figure S1a; 1991–2005) show a further decrease in freshwater availability over parts of the West, Northwest, Northeast, South, and Upper Midwest. Although the relative changes are positive over parts of the South, Southwest, and Southeast (Fig. 1A), the absolute values of low flows are negative, indicating these regions are projected to be drier. There were no changes in low flows over the West North Central regions in 2020 s (2006–2025); however, water scarcity in the region will further enhance by 2030s (2021–2035).

Spatial patterns of current estimates (1991–2005) of high stream temperature (90^th^ percentile) show warming patterns over most of the gauge stations (Figure S2a). The projected changes in high stream temperature in the near future (Fig. 1B), relative to current estimates (Figure S2a), are expected to increase over most gauge stations; the maximum increase will happen over the Northeast, Central, and Upper Midwest regions (Figure S3). Stream temperatures are projected to exceed 27 °C (a critical limit over which water is usually not suitable for cooling; see refs 9 and 30 for details) at most of the gauge stations in the South, Southeast, Northeast, and Upper Midwest regions (Figs 1B and S2a). Surface air temperature, which directly affects evapotranspiration rates (and hence water availability) and water temperature, shows warming patterns (Figure S4) over regions that are projected to become dry (Figs 1A and S1a). The projected changes in surface air temperature are more than 1.4 °C during 2030s over several regions especially in the Northeast, Midwest, and Southwest (Fig. 1C).

### Association between warmer and scarcer water

**Figure 2:**
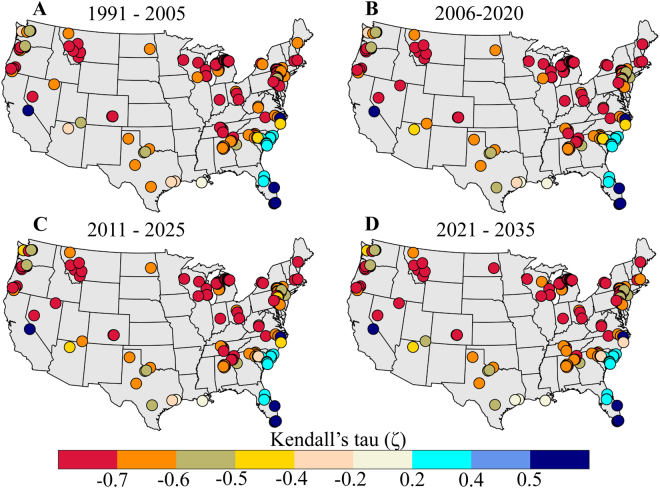
Association between indicators of water stress. (**A**–**D**) Correlation coefficient between monthly surface runoff and stream temperature as measured by Kendall’s tau at 145 USGS gauge stations for **(A)** current (1991–2005) and (**B**–**D**) future time horizons (2006–2035). Correlations are statistically significant at 5% at all gauge stations for both current and future periods. Maps are generated using MATLAB 2015a (Version 8.5, http://www.mathworks.com). Finally, all these maps are organized and labelled in Adobe Photoshop CS Desktop (Version 5.1, https://www.adobe.com) [Software].

There is a strong dependence, as measured using Kendall correlation coefficient (also called, Kendall’s tau^57^; see Methods), between current and future estimates of freshwater availability and stream temperature (Fig. 2). Kendall’s tau is a rank-based measure of association and is resistant to small number of outliers in the data. It measures the strength of monotonic relationship including nonlinear. Its value lies between −1 and 1, inclusive; the maximum positive (negative) value indicates perfect association (disassociation). The Kendall’s tau values of about 0.7 or above (and −0.7 or below) indicate strong dependence^57^. Here Kendall’s correlation coefficient is less than −0.7 at more than 55 gauge stations (out of 145) spread over US during current (Fig. 2A) and future (Fig. 2B–D) time horizons; it shows strong negative dependence between freshwater availability and stream temperature. We observe positive correlation over a few gauge stations in the Southeast coast. Furthermore, the dependence is robust as the correlation values are statistically significant at 5% at all gauge stations for all time horizons. The dependence structures remained unchanged over time (2006–2035 versus 1991–2005) at most of the stations; however, we note the dependence tends towards negative over a few locations. Negative correlations can result from two types of opposite associations: low flows, high stream temperatures (scarcer and warmer water) and high flows, low stream temperatures (wetter and cooler water); the former represents a situation for potential water problems for cooling. Positive correlations can result from two types of direct associations: low flows, low stream temperatures (scarcer and cooler water) and high flows, high stream temperatures (wetter and warmer water); both situations (low flows in the first case and high stream temperatures in the second) can potentially result in water problems for power production.

**Figure 3:**
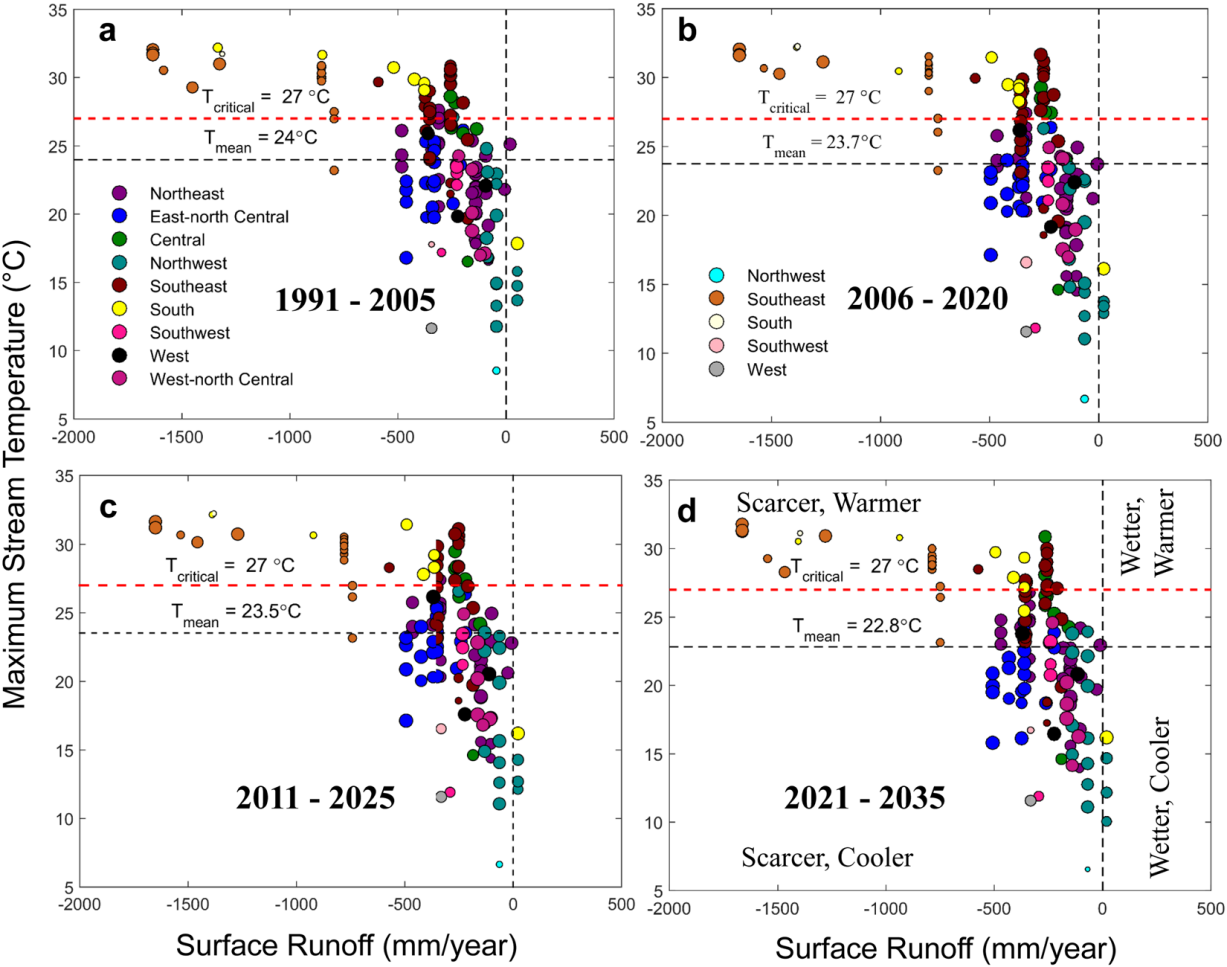
Scatter diagrams between indicators of water stress. (**a**–**d)** Scatter plots showing the relationship between mean surface runoff and maximum stream temperature for nine climatologically homogeneous regions, each shown by different colors, for current (1991–2005) and future time periods (2006–2035). The size of the color-filled circles represents strength of the association, as measured by Kendall’s tau (shown in Fig. 2), between surface runoff and water temperature. For a given region, nature of the association is captured by the different shades of the color; darker (lighter) shades or negative (positive) values of Kendall’s tau represent inverse (direct) relationship. The two dotted horizontal lines are drawn at the ensemble mean of stream temperatures and a critical water temperature limit of 27 °C (a limit over which water is not suitable for cooling; it is ~5 °C lower than the Environmental Protection Agency [EPA] prescribed maximum allowed temperature of ~32 °C). The vertical dotted line is drawn at no flow. The left side of the vertical line represents water scare situations, and the side above 27 °C represents warmer. Each of the scatter plots is divided into four quadrants: scarcer, warmer (top left); scarcer, cooler (bottom left); wetter, warmer (top right); and wetter, cooler (bottom right) as shown in (**d**). The partitioning of the scatter diagrams explicitly identify regions with hot spots – a combination of low flow and high temperature. The figure legend at (**a**) indicates negative values of Kendall’s tau whereas at (**b**) shows positive values. Legends are same for all panels. Figures were generated using MATLAB 2015a (Version 8.5, http://www.mathworks.com). Finally, all these figures are organized and labelled in Adobe Photoshop CS Desktop (Version 5.1, https://www.adobe.com) [Software].

We show scatter plots between mean surface runoff and maximum stream temperature (Fig. 3) to discern the four possible types of relationship as discussed above. Each of the scatter plots has been partitioned into four quadrants by drawing a vertical line at no flow and a horizontal dotted line at 27 °C (a critical threshold for stream temperature over which water is not suitable for cooling^9,30^). Each quadrant of the scatter plots represents a combination of surface runoff (scarcer or wetter) and water temperature (warmer or hotter) as shown in Fig. 3d. The points on the scatter plot represent a pair of values of mean surface runoff and maximum stream temperature at each of the USGS gauge stations. The points are distinguished in nine categories; each represents one of the nine climatologically homogeneous regions^56^ of the mainland US (Figure S3). Most points fall within two quadrants: scarcer - warmer (negative correlation) and scarcer - cooler (positive correlation) – indicating potential water problems especially due to diminished supply (Fig. 3). Most of the gauge stations in the South, Southeast, and Central regions fall in the top left quadrant (scarcer - warmer scenario), with highest concentration of points for Southeast region (indicated by the orange bubbles on the scatter plot) over the next three decades. The spread of the points in the scatter plots remains unchanged over time.

### Multivariate Standardized Water Stress Index (MSWSI)

Water scarcity alone can impact operations of a power plant. On the other hand, even when abundant water resources are available but if the available water is too hot to be used for cooling, power production will be affected. However, a distress situation could happen when both conditions – water scarcity and high stream temperature - happen concurrently. We showed strong dependence between warmer and scarcer water above. Here we develop a new dimensionless standardized water stress index (SWSI) to capture the joint effects of warmer and scarcer water in a single metric and employ this metric to assess the vulnerability of thermoelectric power production due to water problems. Subsequently, first, we compute empirical joint probability distribution of monthly surface runoff and monthly stream temperature. Next, we transform the cumulative probability to derive the SWSI using inverse of standard normal distribution (See Methods).

**Figure 4:**
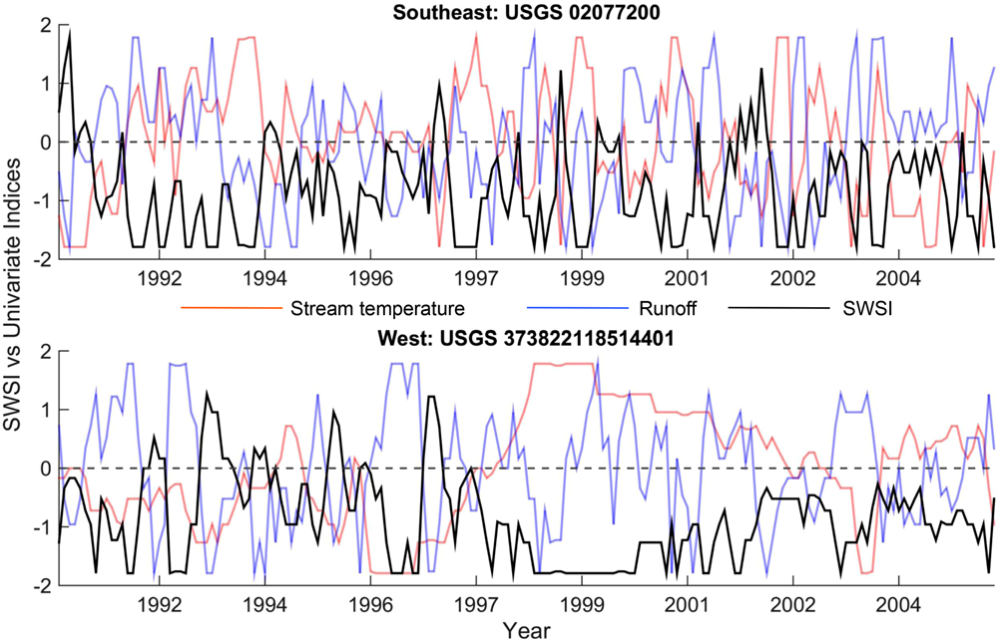
Time series comparison of SWSI relative to univariate water stress indices. Sample time series of 3-month SWSI is compared with standardized low surface runoff flow and high stream temperature indices at 3-month time scale. The top and bottom panel shows selected USGS gauge locations over Southeast (North Carolina, USGS Station ID 02077200, latitude 36.39° and longitude 79.20°) and West (California, USGS Station ID 373822118514401, latitude 37.64° and longitude 118.86°). Figures are generated using MATLAB 2015a (Version 8.5, http://www.mathworks.com) [Software].

**Figure 5:**
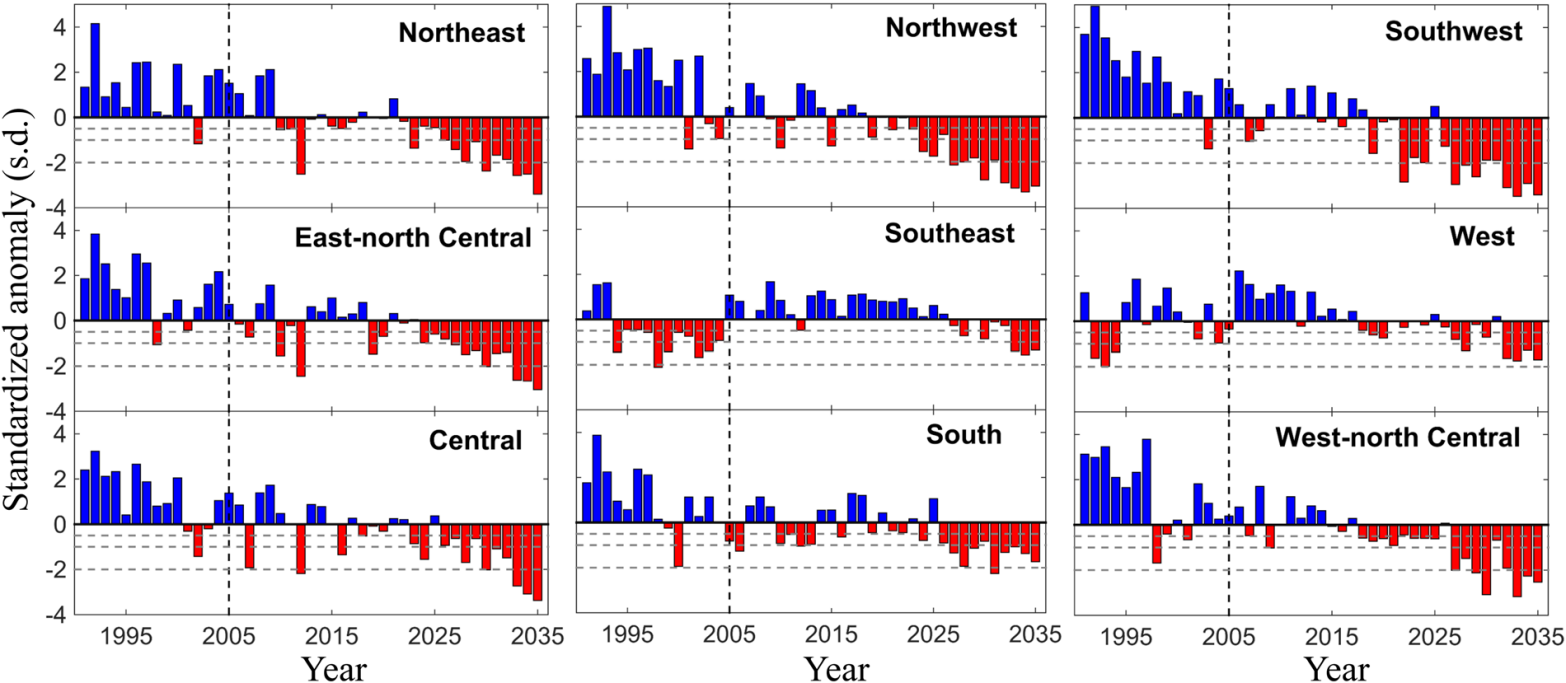
Spatial trend of Standardized Anomaly of SWSI. Time series of standardized anomaly for each of the nine climatologically homogeneous regions for 45 years (1991–2035). Years that are water stressed (negative values of standardized anomaly) are shown in red. The horizontal dashed lines are drawn at −0.5, −1.0, and −2.0 Standard Deviations (SDs) to indicate three water stress levels: 0.5-, 1-, and 2-SD. The vertical line demarcates current and future time periods. Figures are generated using MATLAB 2015a (Version 8.5, http://www.mathworks.com). Finally, all these figures are organized and labelled in Adobe Photoshop CS Desktop (Version 5.1, https://www.adobe.com) [Software].

Figure 4 compares 15-year record of SWSI from January 1991 to December 2005 at a moving window timescale of three-month with univariate water stress indices (low surface runoff and high stream temperature) computed at the same time scale for the two selected stream gauge locations (at Southeast: North Carolina and West: California). As shown in the figure (Fig. 4), the SWSI is able to simulate the persistence resulting from low water availability and onset of high stream temperature (for instance, during 1997–2001 in West in which stream temperature was significantly high for the whole period while signatures of low flows are evident in the years 1998 and 2001). To envisage annual variations of water stress, for each year, we compute Standardized SWSI anomaly; thus, expressing the SWSI as distance from its long-term mean in terms of its SDs (See Methods). Figure 5 presents spatially averaged time series of Standardized SWSI anomaly for all nine regions, which indicates annual water stress at different stress levels (−0.5, −1 and −2 SD levels; in which −0.5 being the moderate and −2 is the extreme scenario). The interannual variability of the standardized anomalies for all nine regions shows that most of the years in the next three decades would be water stressed (Fig. 5). In general, except Southeast and West, in all regions after 2025, are most likely to exceed −2 SD water stress (Fig. 5) level. For Southeast and West, we note a persistent trend in water stress in present-day condition (during 1991–2005) as indicated by red bars below mean in the anomaly time series, which is likely to be continued in 2030s. The number of years in the future exceeding −2 SD risk level is highest in the Northwest and Southwest US.

### Estimates of regional power production at risk

**Figure 6:**
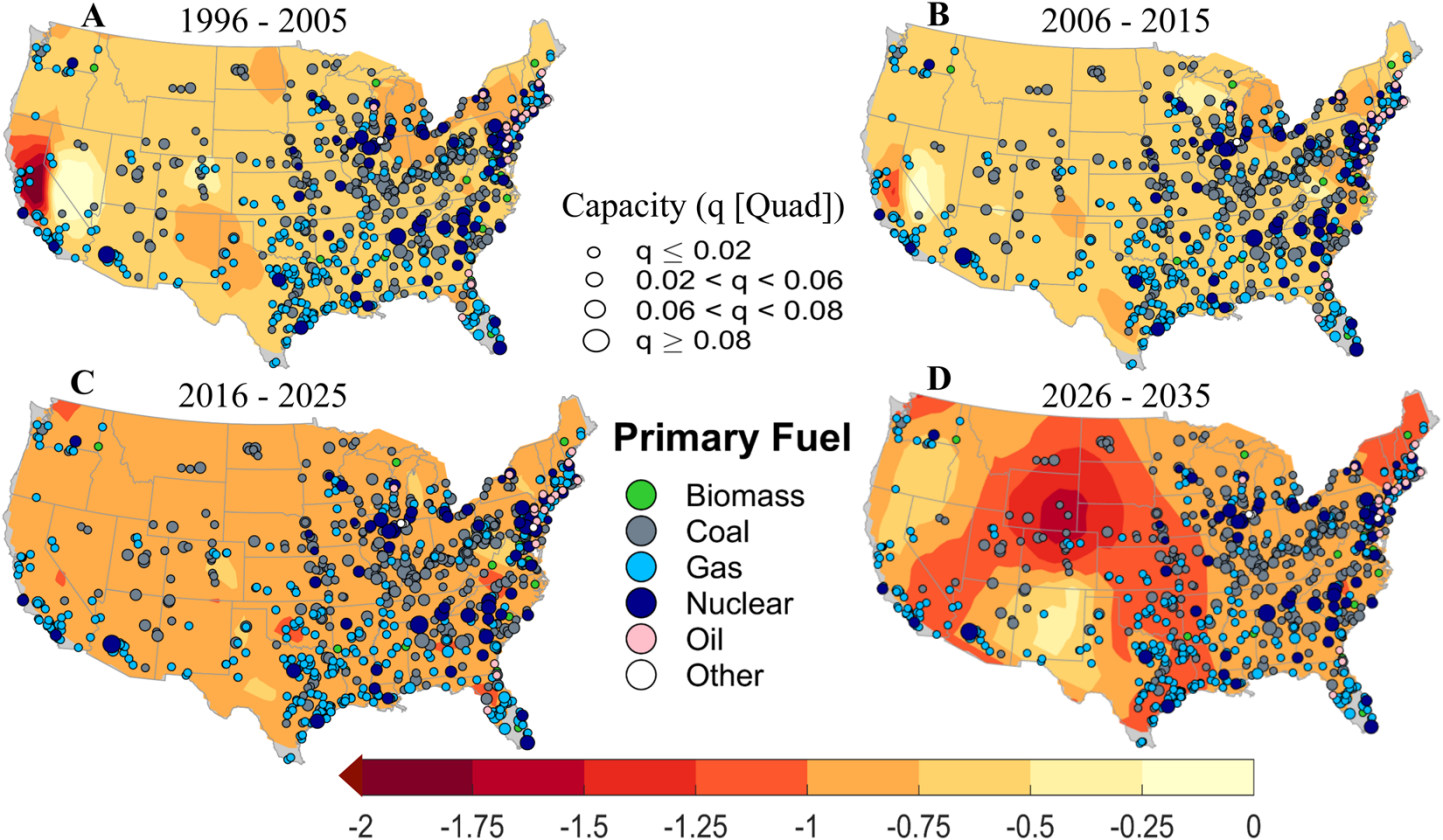
Contours of standardized water stress index. (**A**–**D**) Spatial location, installed power production capacity (in Quad), and primary fuel types of thermoelectric power plants superimposed over contours of decadal mean of standardized water stress index for current (1996–2005) and future (2006–2035) time periods. Size of the filled color circle is directly proportional to the installed production capacity. Different shades of water stress contours indicate risk level due to the joint effects of low flow and high stream temperature. Grey shades in the map indicate regions where data is not available. Figures are generated using MATLAB 2015a (Version 8.5, http://www.mathworks.com). Finally, all these maps are organized and labelled in Adobe Photoshop CS Desktop (Version 5.1, https://www.adobe.com) [Software].

We show filled contours of SWSI for current (1996–2005; Fig. 6A) and future (2006–2035; Fig. 6B,C) time horizons to visualize the spatial patterns of water stress over different regions. For each time horizons, we draw the contour using decadal mean of the monthly SWSI. Spatial variations of the SWSI contours indicate intensification of water stress in the future, especially in 2030s (Fig. 6D), and hence potentially increased vulnerability to thermal power production. To get a qualitative sense of how much power production will be at risk and which thermal power stations would be affected, we superimpose the attributes of power plants such as their locations, annual production capacity, and sources of primary fuels on the contour maps. In this study, we have considered only wet-cooled thermal power plants with total annual capacity of 11.07 Quad (Table S3), of which coal-fueled and nuclear plants generate about 5.89 and 2.74 Quad respectively. Most of the thermoelectric power plants - especially nuclear-fueled, which requires more water for cooling than others – are concentrated in the Northeast, Southeast, South, Central, and East North Central. Overall, these regions are projected to be the most water stressed (Fig. 6D).

**Figure 7:**
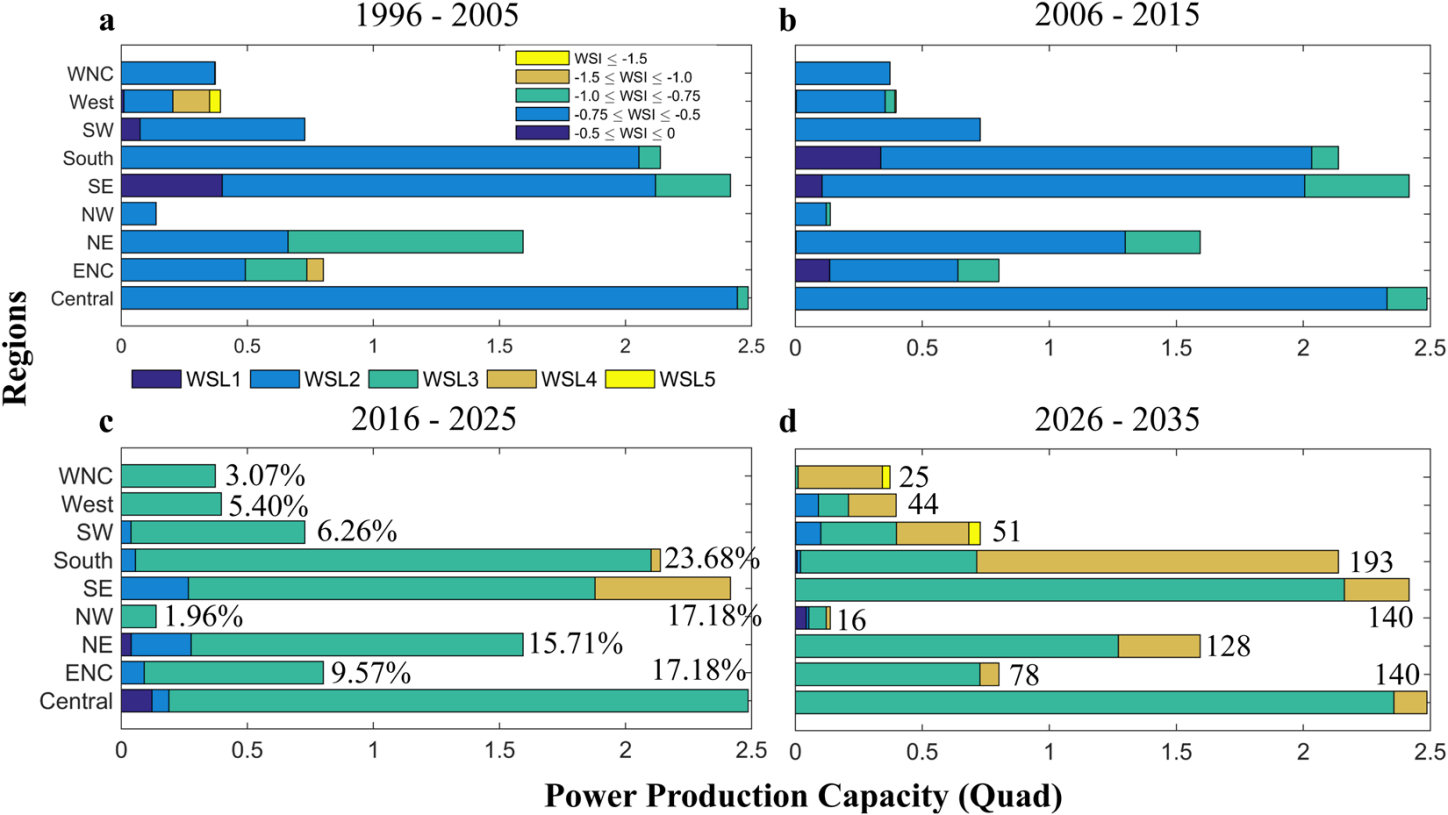
Regional distribution of power production at risk under various water stress levels (WSL). (**a**–**d**) Bar plots showing the breakup of power production at risk for five different water stress risk levels over nine regions for (**a**) current (1996–2005) and (**b**–**d**) future (2006–2035) time periods. The annual power production capacity for each region is shown in (**c**). The total production capacity is 11.07 Quad (Table S3). The number of power plants in a specific region is shown in (**d**). The total number of power plants is 815 (Table S3). Five water stress levels (WSL) are defined as follows: WSL1 (−0.5 ≤ WSI ≤ 0), WSL2 (−0.75 ≤ WSI ≤ −0.5), WSL3 (−1.0 ≤ WSI ≤ −0.75), WSL4 (−1.5 ≤ WSI ≤ −1.0), and WSL5 (WSI ≤ −1.5), where WSI stands for water stress index. WSL1 (WSL5) indicates the less (most) severe condition. WNC: West North Central, SW: Southwest, SE: Southeast, NW: Northwest, NE: Northeast, and ENC: East North Central. Figures are generated using MATLAB 2015a (Version 8.5, http://www.mathworks.com). Finally, all these figures are organized and labelled in Adobe Photoshop CS Desktop (Version 5.1, https://www.adobe.com) [Software].

To describe the changing nature of water stress level (WSL) over time, we categorize the mean standardized Water Stress Index (WSI) into five levels (Fig. 7a) with level 1 (*WSL1*: −0.5 ≤ *WSI* ≤ 0) being the least and level 5 (*WSL5*: *WSI* ≤ −1.5) being the most severe stress condition.

We use this classification to estimate how much power production will be impacted for each of the nine regions over the next three decades. Currently (Fig. 7a), a large proportion of production capacity (79%) is exposed to water stress level 2 (*WSL2*: −0.75 ≤ *WSI* ≤ −0.5) in all regions (Table S4); in the Northeast, more than half (58%) of the installed production capacity is exposed to water stress level 3 (*WSL3*: −1.0 ≤ *WSI* ≤ −0.75). By 2020 s (Fig. 7c), 86.5﻿% of production capacity is exposed to WSL3 across all regions (Table S4). By 2030s (Fig. 7d), 70% of annual production capacity (Central, East North Central, Northeast, Northwest, Southeast, and Southwest) is exposed to WSL3 and 27% (South, Southwest, West, and West North Central) is exposed to water stress level 4 (*WSL4*: −1.5 ≤ *WSI* ≤ −1.0). Here we show that as the water stress intensifies due to climate change, power production would be impacted in the South, Southwest, West, and West North Central regions by 2030s (Fig. 7d).

## Discussions

We developed a new metric to characterize water stress and applied it to study the vulnerability of thermoelectric power production over the contiguous United States for the next three decades under nonstationary^58,59^ climate. The metric can be applied to assess impacts of climate extremes in other sectors, especially in scenarios in which the simultaneous occurrence of two or more climate stressors can cause severely adverse effects than had the stressors are considered alone. One of the major challenges of climate impacts assessment at near-term (0–30 year) horizons is the consideration of *deep uncertainty* ^13^ in climate projections. Here deep uncertainty refers to internal variability, which is inherent to the climate system, or multi-model response variability, which results from limitations of our understanding of physical processes and numerical models of the climate system. Here we considered deep uncertainty by including projected hydro-meteorological data from all plausible alternative future climates. Furthermore, climate projections are less credible at the scale of the location of power plants; statistical downscaling of climate variables can be performed to get reliable estimates. Other alternative options would be to run physically based hydrological models. However, both approaches – statistical downscaling and hydrological modeling – would introduce additional uncertainties in addition to uncertainty from climate projections.

In the twentieth century, the United States had abundant freshwater resources to be used for cooling thermoelectric power plants. However, with growing population, increasing water demands from other sectors, changing precipitation patterns, and increasing evaporation rates, several regions within US have started to experience water shortage – and that too during dry and hot summers. Demand for electricity in US is expected to grow by 29% from 2012–2040 (EIA, 2014)^10^. Developing alternative energy portfolios would take some time; nevertheless, transformative cooling technologies can be developed that would require less water and can operate at full capacity even when available water would be warmer than the design threshold. One option for reducing the vulnerability of electricity production depending on the availability of water would be to replace the cooling system with a less water intensive one such as by replacing a circulation system with a hybrid or a dry cooling system. In addition, we should continue investing in alternative energy sources to reduce our reliance on thermal energy; this would also facilitate in climate mitigation.

A few caveats should be considered so that the results presented here are not over-interpreted. The reader is pointed to a discussion of various implicit hypotheses and/or assumptions in the discussions prior to the results section. Furthermore, in this study, we have not considered other stressors such as multi-sectors water demand, population growth and demographic shifts, technological advancements, changes in life styles, and regulatory issues. The efficiency of thermoelectric power plant will likely to impacted by operational factors, such as, part-load or transient operation, regulation, and design and maintenance issues^60^; however, these considerations are beyond the scope of present analysis. Here we solely focus on key environmental stressors regulating the plant efficiency, with the implicit assumption that the other factors do not change. Future studies may need to scrutinize these assumptions carefully. Finally, projections of changes in climate stressors such as water scarcity and stream temperature exhibit various tradeoffs in terms of uncertainty over time horizons. At multi-decadal to century time scales, the dominant source of uncertainty is expected to result from our inability to credibly project greenhouse-gas emissions scenarios, which are treated as plausible what-if trajectories and are not based just on physics but also on various human and natural factors. In the near term from interannual to a couple of decades in the future, the uncertainties are dominated by the natural variability in the climate system (which are comparable to the change signal at these time horizons) including initial condition variability (i.e., the intrinsic variability of the climate system even when the physical assumptions are assumed to be identical: this uncertainty is generated by the complex nonlinear dynamics of the climate system and in the near-term can compare with multi-model ensemble variability). Based on all these considerations, this study and the results presented herein should be viewed as analogous to sensitivity analysis. The primary focus here is on changes in water stress along with its uncertainties in anticipation of future changes, and especially the potential impacts on power production due to climate change alone by keeping other stressors relatively invariant.

## Methods

### Estimation of Freshwater Availability

Freshwater availability or surface runoff is estimated as the difference between precipitation (*P*) and evapotranspiration (*E*)^11–13,41,49^; this approach has been used extensively in the literature, and the quantity *P-E* has been termed as “available precipitation”^11,12,41^, or “available freshwater”^13,49^ or “atmospheric moisture budget”^48^. Monthly precipitation and evapotranspiration (includes evaporation from underlying surface and vegetation) data were retrieved from the latest generation of global climate models (GCMs) participating in the Coupled Model Intercomparison Project phase 5 (CMIP5)^50^. While excess evapotranspiration over precipitation indicates regional water scarcity, it may not be a true indicator of water availability as we are not considering contributions from groundwater and inter-basin transfers^11,13,49^. Past and future precipitation (*pr*) and evapotranspiration (*evspsbl*) data were obtained for “historical”^50^ and “representative concentration pathways (RCPs)”^50,51^ experiments, respectively. We have focused our analysis over contiguous United States (excluding Alaska and Hawaii) over the next three decades (2006–2035); future data in CMIP5 archive are available from 2006 onward. Over near-term (0–30 years) projection horizons, the trend of climate change is comparable to climate uncertainty; hence, before we proceed, it is important to understand the relative contributions of different sources of uncertainty in climate projections.

Uncertainty in climate projection stems primarily from three sources: multi-model spread or model response variability (MRV), climate internal variability (CIV), and emissions scenario uncertainty. MRV represents lack of our understanding of atmospheric processes and inadequate numerical modeling of these processes in climate models; different models initialized with the same initial conditions give disparate response to the same forcing. CIV is the natural fluctuations of the climate system in the absence of any external forcing, and it represents sensitive dependence on initial condition. Scenario uncertainty results from the insufficient information about future emissions. The different sources of uncertainty dominate at different time horizons; specifically, MRV and CIV dominate at decadal to multi-decadal scales (0–30 years). The dominance of MRV and CIV is more pronounced at regional scales. CIV dominates until about 2040 s, and MRV and scenario uncertainty dominates after that until the end of the century (IPCC Working Group I’s Fifth Assessment Report; Chapter 11; Figure 11.8^61^). Uncertainty in climate projections, collectively, is also referred as *deep uncertainty* ^13^.

A general practice in climate impacts assessment has been to consider MRV by including simulations from multiple models. CIV^37–39,62^, as discussed, dominates climate uncertainty in the first few decades and at regional scales. Hence, a priori assumption on the number and selection of climate models and exclusion of initial condition runs limits the opportunity to explore the full range of climate uncertainty in future estimates of water stress especially in a nonstationary^58,59^ environment. In order to account for CIV, we consider past and future climate data from all available initial condition (IC) runs in the CMIP5 archive. To account for deep uncertainty in climate projections, we have considered climate data from all available GCMs, RCPs, and ICs combinations. The name of climate models along with their horizontal spatial resolutions and the number of initial condition runs used for historical and RCP scenarios is summarized in Table S1. In this study, we have used climate data from 45 models, 4 RCPs (RCP-2.6, 4.5, 6, and 8.5), and 475 ICs (159 for historical and 316 for RCPs) to estimate freshwater availability. Both precipitation and evapotranspiration data were bi-linearly interpolated from models’ native grid (Table S1) to a common grid of 2-degree spatial resolution using Climate Data Operators (CDO) software (https://code.zmaw.de/projects/cdo) before computing freshwater availability at each of the interpolated grid point (2-degree). Current estimates of surface runoff are obtained for 1991–2005. The projected changes in freshwater availability, relative to current estimates, are computed for three future time horizons: 2006–2020, 2011–2025, and 2021–2035. We have not employ any hydrological model in this study since these models are based on simplified assumptions of physical and unobserved heterogeneous sub-surface processes, which are often modeled using a large number of parameters. Hydrological models are calibrated for a specific river basin or watershed; the calibration may not be appropriate to simulate hydro-climatic processes in the future especially under nonstationary climate. Hydrological models may give relatively precise estimates of upstream and downstream flow; however, the overwhelming uncertainty resulting from the calibration and heavy use of parameterization might undermine its precision. Furthermore, hydrological models are computationally time intensive to run, which restricts its utility to run them only with a few climate model outputs and may be with just one initial condition run. A priori selection of subset of climate models and exclusion of initial condition runs (important at near-term horizons as CIV dominants uncertainty in climate projections) will limit the opportunity to explore the full range of uncertainty and may not be appropriate to estimate low values (such as 10^th^ percentile) of surface runoff at 0–30 years.

### Prediction of Stream temperature

Unlike precipitation and evapotranspiration, stream temperature data are not directly available from CMIP5 models. We predict stream temperature using support vector regression models^63,64^, which are developed using observed stream temperature at United States Geological Survey (USGS) gauge stations and 2-meter surface air temperature (*tas*) from CMIP5 models. We obtained monthly stream temperature data at 145 USGS gauge locations (http://waterdata.usgs.gov/nwis) across 18 hydrologic units (https://water.usgs.gov/GIS/huc.html), identified by a unique hydrologic unit code (HUC), from contiguous US based on maximum data availability. We did not consider stations with more than 7-years of missing record. Over most of the gauges, stream temperature data are available from 1996–2013. There are several stations over which data are missing during 1991–2005. We filled the missing stream temperature record using three approaches: (i) Missing values prior to 1996 is hind-casted using support vector regression (SVR) models with surface air temperature as predictor, (ii) If the stream temperature data for any month are missing between 1996–2005, they are estimated from neighboring stations within that hydrologic units using a regularized expectation-maximization algorithm^65^, and (iii) Stream temperature of those stream gauges with no neighboring stations within that hydrologic units (such as for HUC-6, 8, 9,14, and 15) are imputed using time series interpolation technique with a shape-preserving piecewise cubic polynomial function^66,67^.

We use 50^th^ percentile of all climate realizations (Table S2) of surface air temperature (*tas*) as a predictor for the SVR model to simulate monthly mean stream temperature. Data from GCMs are available at coarser spatial resolutions (Table S1) and hence, are not credible to use as predictors at the spatial resolution of stream gauge location. One of the standard practices is to downscale^68^] the coarse resolution climate data to the desired finer spatial scales. We retrieved (statistically) downscaled surface air temperature data at a spatial resolution of 0.125 degrees (~9 miles) for all available GCMs, RCPs, and ICs combinations (Table S2) from the following source: http://gdo-dcp.ucllnl.org/downscaled_cmip_projections/dcpInterface.html. The Bias-Corrected Statistical Downscaling (BCSD)^69^ methodology was used to downscale the archived data. The list of climate models (37 GCMs) along with the number of initial condition runs for historical and RCPs experiments for the downscaled surface air temperature data is summarized in Table S2. We have a total of 316 ensembles - 84 historical and 232 RCPs. The number of climate models and total number of initial condition runs are different for the downscaled temperature data (Table S2) from that of precipitation and evapotranspiration (Table S1).

We develop a least squares support vector regression (LS-SVR)^63^ model at each USGS gauge station to predict future stream temperatures. The functional dependence between stream temperature (response variable) and air temperature (predictors) is developed based on the correlation between monthly stream temperature and 2-m surface air temperature^55,70,71^, and we assume that the same relation will hold good in the projected time scales. Further, we included lagged values (one and two months before current month) of surface air temperature to consider seasonality effects. We use 180 months (1991–2005) of data to train the LS-SVR model and 96 months (2006–2013) for validation. The computation is performed within commercially available software MATLAB using the StatLSSVM^64,72^ package. The performance of the models is assessed using the Nash-Sutcliffe Efficiency (NSE)^73^ index and Pearson’s linear^57^ correlation coefficient (Figures S5 and S6). We use ensemble median (50^th^ percentile) surface air temperature from all climate simulations (84 for historical and 232 for future) as predictors to predict the monthly historical and future stream temperature. To characterize water stress due to warmer water, we estimate 90^th^ percentile of predicted monthly mean stream temperature at each gauge station.

### Association between freshwater availability and stream temperature

Based on Shapiro-Wilk test^74^, the normality assumption for the marginal distributions of water supply and stream temperature is rejected (at 5% significance level) at all stream gauge locations; following which, we measure the strength of the association using a rank-based nonparametric correlation measure, Kendall’s tau (ζ). It measures the strength of monotonic relationship including nonlinear and is resistant to a small number of outliers in the data. The values of Kendall’s tau lie between −1 and 1, inclusive; the extreme values represent perfect correlation. Positive (negative) values of Kendall’s tau indicate perfect agreement (disagreement). In this study, a negative value of Kendall’s tau can result from two possibilities: low flows, high stream temperatures (scarcer, warmer) and high flows, low stream temperatures (wetter, cooler); the former case represents a water stress scenario. Similarly, a positive value of Kendall’s tau can result from two possibilities: low flows, low stream temperatures (scarcer, cooler) and high flows, high stream temperatures (wetter, warmer); here either case (low flow in the first and high stream temperature in the second) can lead to water stress. To discern the particular relationship, we visualize the data using scatter diagrams between water availability and stream temperature. The scatter plot is divided into four quadrants by drawing one vertical line at no flow and one horizontal dotted line at 27 °C (a critical limit for stream temperature above which water is not suitable for cooling; refer to ref.^24^ for details), respectively.

### Multivariate Standardized Water Stress Index (MSWSI)

To capture the joint effects of warmer and scarcer water in a single dimensionless metric and to assess the vulnerability of thermoelectric power production, we develop a new nonparametric standardized bivariate water stress index. The methodology has been adapted from the approach used to develop indices for drought characterization such as the Standardized Precipitation Index (SPI)^75^. Further, the index is motivated from multivariate characterization of droughts, in which both parametric^42,43^ (copula-based) and nonparametric^44,45^ (empirical distribution using plotting position) joint probability distributions are considered in the literature. However, without assuming a specific distribution of covariates, here we derive joint distribution of low surface runoff and high stream temperature using rank-based Gringorten plotting position^76^ formula as, *P*_*j*_(*w*_*k*_, *t*_*k*_) = (*I*_*k*_ − 0.44)/(*N* + 0.12), where *I*_*k*_ is the number of occurrences of the pair (*w*_*i*_, *t*_*i*_) for which *w*_*i*_ ≤ *w*_*k*_ and *t*_*i*_ ≥ *t*_*k*_, where, *N* is the number of observation, *w* and *t* indicate freshwater availability and stream temperature at an accumulated time scale of three-month (*n* = 3) to facilitate temporal analysis of water stress. The marginal probability is obtained using univariate form of Gringorten plotting position formula, expressed as, *P*(*y*_*i*_) = (*i* − 0.44)/(*N* + 0.12), where *i* is the rank of the observed low flow and stream temperature time series accumulated at a time scale of three-month and arranged in the ascending (*w*_*i*_ ≤ *w*_*k*_) and descending (*t*_*i*_ ≥ *t*_*k*_) order respectively. Subsequently, the joint cumulative probability is transformed to derive the standardized water stress index (SWSI) using the inverse of standard normal distribution as, *SWSI* = *ϕ* ^−1^(*P*_*j*_), where *ϕ* is the standard normal distribution function and *P*_*j*_ is the joint probability computed above. First, SWSI is computed as a moving window of three-month and at each stream gauge station to capture seasonality of combined water stress emerged from low flows and high stream temperatures. Next, annual SWSI is computed by taking mean of monthly values for each year. Finally, annual SWSI is expressed as standardized anomaly, which is computed as the magnitude of SWSI anomaly (departure from its long-term mean), divided by the standard deviation (SD) of the detrended (linear^57^) SWSI series. Thus, water stress for each year is expressed as distance from mean of the standardized anomaly in terms of SD. To characterize regional water stress, we compute area-weighted (weight is calculated as cosine of the latitude of gauge station) spatial average of standardized anomaly for each of the nine regions for each year from 1991–2035. A year is considered as water stressed, if standardized anomaly for that year is negative. Furthermore, we define two risk levels, namely 1-SD and 2-SD (similar metric was defined in ref.^46^ to characterize droughts), depending on the value of anomalies exceeding −1.0 SDs and −2.0 SDs, respectively.

We draw filled contours of decadal mean of monthly SWSI at different stress levels to characterize the spatial variations of water stress. The filled contours help us visualize the risk profiles over different regions. In addition, it helps us understand which regions are projected to be the most water stressed, and hence power plants in those regions will potentially be most likely affected. To quantify regional power production at risk, we define five water stress levels (WSL) using SWSI based on US Drought Monitor (DM) Classification Scheme^77^ as follows: WSL1 (−0.5 ≤ *WSI* ≤ 0), WSL2 (−0.75 ≤ *WSI* ≤ −0.5), WSL3 (−1.0 ≤ *WSI* ≤ −0.75), WSL4 (−1.5 ≤ *WSI* ≤ −1.0), and WSL5 (*WSI* ≤ −1.5), where *WSI* stands for water stress index. WSL1 indicates the abnormally dry condition (D0, representing 20–30^th^ percentile value of SPI in USDM classification scheme) and WSL5 indicates the extreme condition (D3, indicating 2–5^th^ percentile value of SPI in USDM classification scheme)^77^. We show a break up of annual production capacity affected due to different water stress levels for each of nine regions from 1996–2035.

### Power plants data

Spatial locations of thermoelectric power plants and their annual installed capacity were taken primarily from two sources: the Electric Power Research Institute (EPRI, 2011)^5^ and the Energy Information Administration database (EIA, 2013)^78^. We have considered 815 thermoelectric power plants with a total annual production capacity of 11.07 Quad (Quadrillion British Thermal Units [QBTU]; in short Quad). We have reported our results in Quad, a unit commonly used by U.S. Department of Energy and U.S. Energy Information Administration (EIA). To put the energy unit in perspective, one QBTU would provide all of the energy demand for New York State for approximately three months (Source: Resource Revolution: Meeting the world’s energy, materials, food, and water needs, Nov 2011, The McKinsey Global Institute). The regional distribution of production capacity and the number of power plants are summarized in Table S3. The coal-fueled and nuclear power plants contribute about 5.90 and 2.74 Quad, respectively.

Two of the most common types of cooling systems used in thermoelectric power plants are: once-through (withdraws water from near-by sources) and wet-recirculating (withdraws water only to replace the water lost to evaporation and it reuses the cooling water). Once-through cooling system uses large amounts of water and has greater potential to affect the aquatic life and ecosystems negatively than that of the other cooling systems. Relative to once-through cooling systems, the recirculating systems withdraw less water (~5%)^79^ but consume more water through evaporation and drift^80^. Most power plants built prior 1970 use open-loop system, in which water is withdrawn from a water source, used to condense steam for cooling and then discharged back to the source^80^. Further, review of literature suggests that^79^, fossil fuel and nuclear power plants withdraw water in an increasing order and wet-recirculating systems use more water per MWh than once-through system. Therefore, in a growing concern for water vulnerability we consider both once-through and wet-circulating cooling systems, which collectively inferred as wet-cooled system in this study.

## Electronic supplementary material

US Power Production at Risk from Water Stress in a Changing Climate
