# Author Correction: US Power Production at Risk from Water Stress in a Changing Climate

**DOI:** 10.1038/s41598-018-24600-y

**Published:** 2018-04-18

**Authors:** Poulomi Ganguli, Devashish Kumar, Auroop R. Ganguly

**Affiliations:** 1 Sustainability and Data Sciences Laboratory (SDS Lab), Department of Civil and Environmental Engineering, Northeastern University, Boston, 02115 Massachusetts United States; 2 Present Address: GFZ German Research Centre for Geosciences, Section 5.4 Hydrology, 14473 Potsdam, Germany

Correction to: *Scientific Reports* 10.1038/s41598-017-12133-9, published online 20 September 2017

This Article contains typographical errors.

In the first sentence of the Introduction section,

“Presently, 91% (3500 million MW h)^1^ of total electricity in the United States is generated by fossil-fueled thermoelectric power plants, which use 45% (~161 billion gallons per day)^2^ of total freshwater withdrawal (the single largest use of fresh water), 90% of which is used for cooling.”

should read:

“Presently, 91% (3500 million MW h)^1^ of total electricity in the United States is generated by thermoelectric (fossil-fuel and nuclear) power plants, which in turn utilize 45% (~161 billion gallons per day)^2^ of the total water withdrawal, 90% of which is used for cooling.”

