# Supplementary material for: US Power Production at Risk from Water Stress in a Changing Climate
Source: Sci Rep. 2017 Sep 20;7:11983. doi: 10.1038/s41598-017-12133-9 (PMC5607259; doi:10.1038/s41598-017-12133-9)
Supplement: Supplementary file 1 — US Power Production at Risk from Water Stress in a Changing Climate [file 41598_2017_12133_MOESM1_ESM.pdf]

Supplementary Materials for

US Power Production at Risk from Water Stress in a Changing Climate

Poulomi Ganguli, Devashish Kumar, and Auroop R. Ganguly*

Sustainability and Data Sciences Laboratory (SDS Lab),
Department of Civil and Environmental Engineering,
Northeastern University, Boston, MA-02115, USA
Email: a.ganguly@neu.edu
Phone: 617-373-6005

Table S1. List of CMIP5 climate models along with their horizontal resolutions (Longitude x Latitude) and the number of initial condition runs used for historical and RCPs experiments

Table S2. List of CMIP5 models for which downscaled surface air temperature is available along with the number of initial conditions for historical and future experiments

Table S3. Regional thermoelectric power production capacity

Table S4. Regional annual power production capacity (Quad) under various water stress levels (WSL)

Fig S1. Current surface runoff. a,b, Spatial patterns of current (1991-2005) **(a)** low (10th percentile of all climate simulations) and **(b)** median (50th percentile) surface runoff. Figures are generated using MATLAB 2015a (Version 8.5, URL: http://www.mathworks.com).

Fig S2. Current stream temperature. a,b, Spatial patterns of current (1991-2005) **(a)** high (10th percentile of all climate simulations) and **(b)** median (50th percentile high stream temperature. Figures are generated using MATLAB 2015a (Version 8.5, URL: http://www.mathworks.com).

Fig S3. U.S. climate regions. Nine climatically homogeneous regions within the contiguous United States used in this study (see ref. 41 for details). ENC: East North Central, NE: Northeast, NW: Northwest, SE: Southeast, SW: Southwest, WNC: West North Central. Figure is generated using MATLAB 2015a (Version 8.5, URL: http://www.mathworks.com).

Fig S4. Current surface air temperature. a,b, Spatial patterns of current (1991-2005) **(a)** high (10th percentile of all climate simulations) and **(b)** median (50th percentile) surface air temperature. Figures are generated using MATLAB 2015a (Version 8.5, URL: http://www.mathworks.com).

Fig S5. Evaluation of performance of SVR models. Performance of SVR in predicting stream temperature at training phase (1991 – 2005) in few selected stream gauges over the nine climatologically homogeneous regions. Figures are generated using MATLAB 2015a (Version 8.5, URL: http://www.mathworks.com).

Fig S6. Evaluation of performance of SVR models. Performance of SVR in predicting stream temperature at validation phase (2006 - 2013) in few selected stream gauges over the nine climatologically homogeneous regions. Figures are generated using MATLAB 2015a (Version 8.5, URL: http://www.mathworks.com).

Table S1. List of CMIP5 climate models along with their horizontal resolutions (Longitude x Latitude) and the number of initial condition runs used for historical and RCPs experiments

No	Model Name	Resolution (lon x lat)	Historical	RCP2.6	RCP4.5	RCP6	RCP8.5
1	ACCESS1-0	192 x 145	2	-	1	-	1
2	ACCESS1-3	192 x 145	3	-	1	-	1
3	BCC-CSM1.1	128 x 64	3	1	1	1	1
4	BCC-CSM1.1(m)	320 x 160	-	1	1	1	1
5	BNU-ESM	128 x 64	1	1	1	-	1
6	CanESM2	128 x 64	5	5	5	-	5
7	CCSM4	128 x 64	8	5	6	6	5
8	CESM1-BGC	288 x 192	1	-	1	-	1
9	CESM1-CAM5	288 x 192	3	3	3	3	3
10	CESM1-FASTCHEM	288 x 192	3	-	-	-	-
11	CESM1-WACCM	144 x 96	7	3	3	-	-
12	CMCC-CESM	96 x 48	1	-	-	-	1
13	CMCC-CM	480 x 240	-	-	1	-	1
14	CNRM-CM5-2	256 x 128	1	-	-	-	-
15	CNRM-CM5	256 x 128	1	3	1	-	5
16	CSIRO-MK3-6-0	192 x 96	10	10	10	10	10
17	CSIRO-MK3L-1-2	64 x 56	3	-	-	-	-
18	EC-EARTH	320 x 160	1	-	2	-	2
19	FIO-ESM	128 x 64	-	3	3	3	2
20	FGOALS-g2	128 x 60	5	1	-	-	1
21	GFDL-CM2p1	144 x 90	2	-	10	-	-
22	GFDL-CM3	144 x 90	5	-	-	1	1
23	GFDL-ESM2G	144 x 90	1	-	1	1	1
24	GISS-E2-H-CC	144 x 90	1	-	1	-	1
25	GISS-E2-R-CC	144 x 90	1	-	1	-	1
26	GISS-E2-H	144 x 90	10	3	15	3	2
27	GISS-E2-R	144 x 90	22	3	13	3	5
28	HadCM3	96 x 73	10	-	10	-	-
29	HadGEM2-CC	192 x 145	8	-	1	-	3
30	HadGEM2-ES	192 x 145	-	4	4	4	4
31	INM-CM4	180 x 120	1	-	1	-	1
32	IPSL-CM5A-LR	96 x 96	6	4	4	1	4
33	IPSL-CM5A-MR	144 x 143	3	3	1	1	1
34	IPSL-CM5B-LR	96 x 96	1	-	1	-	1
35	MIROC4h	640 x 320	3	-	3	-	-
36	MIROC5	256 x 128	5	5	5	5	5
37	MIROC-ESM	128 x 64	3	1	1	1	1
38	MIROC-ESM-CHEM	128 x 64	1	1	2	1	1
39	MPI-ESM-LR	192 x 96	3	2	3	-	3
40	MPI-ESM-MR	192 x 96	3	1	3	-	1
41	MPI-ESM-P	192 x 96	2	-	-	-	-
42	MRI-CGCM3	320 x 160	5	1	1	1	1
43	MRI-ESM1	320 x 160	1	-	-	-	-
44	NorESM1-ME	144 x 96	1	1	1	1	-
45	NorESM1-M	144 x 96	3	1	1	1	1
		**Total**	**159**	**66**	**123**	**48**	**79**

* The short hyphen ('-') indicates particular climate realization is not available for download.

Table S2. List of CMIP5 models for which downscaled surface air temperature is available along with the number of initial conditions for historical and future experiments

No.	Model Name	Historical	RCP2.6	RCP4.5	RCP6	RCP8.5
1	ACCESS1-0	1	-	1	-	1
2	ACCESS1-3	1	-	1	-	1
3	BCC-CSM1.1	1	1	1	1	1
4	BCC-CSM1.1(m)	1	-	1	-	1
5	CanESM2	5	5	5	-	5
6	CCSM4	5	5	4	4	5
7	CESM1-BGC	1	-	1	-	1
8	CESM1-CAM5	3	3	3	2	3
9	CMCC-CM	1	-	1	-	1
10	CNRM-CM5	5	-	1	5	-
11	CSIRO-MK3-6-0	10	10	10	10	10
12	EC-EARTH	4	2	3	-	3
13	FGOALS-g2	3	1	2	-	3
14	FIO-ESM	3	3	3	3	3
15	GFDL-CM3	1	1	1	1	1
16	GFDL-ESM2G	1	1	1	1	1
17	GFDL-ESM2M	1	1	1	1	1
18	GISS-E2-H-CC	1	-	1	-	-
19	GISS-E2-R-CC	1	-	1	-	-
20	GISS-E2-R	5	1	1	1	1
21	HadCM3	4	-	4	-	-
22	HadGEM2-AO	1	1	1	1	1
23	HadGEM2-CC	1	-	1	-	1
24	HadGEM2-ES	4	4	4	4	4
25	INM-CM4	1	-	1	-	1
26	IPSL-CM5A-LR	4	3	4	1	4
27	IPSL-CM5A-MR	1	1	1	1	1
28	IPSL-CM5B-LR	1	-	1	-	1
29	MIROC4h	3	-	3	-	-
30	MIROC5	1	1	1	1	1
31	MIROC-ESM	1	1	1	1	1
32	MIROC-ESM-CHEM	1	1	1	1	1
33	MPI-ESM-LR	3	3	3	-	3
34	MPI-ESM-MR	1	1	1	-	1
35	MRI-CGCM3	1	1	1	-	1
36	NorESM1-ME	1	1	1	1	1
37	NorESM1-M	1	1	1	1	1
	**Total**	**84**	**53**	**73**	**41**	**65**

Table S3. Regional thermoelectric power production capacity

Regions	Number of Power Plants	Annual Production Capacity (Quad)
Central	140	2.487
East North Central (ENC)	78	0.802
Northeast (NE)	128	1.594
Northwest (NW)	16	0.138
Southeast (SE)	140	2.416
South	193	2.138
Southwest (SW)	51	0.728
West	44	0.397
West North Central (WNC)	25	0.373
**Total**	**815**	**11.073**

Table S4. Regional annual power production capacity (Quad) under various water stress levels (WSL)

WSL1: -0.50 ≤ WSI ≤ 0
WSL2: -0.75 ≤ WSI ≤ -0.50
WSL3: -1.0 ≤ WSI ≤ -0.75
WSL4: -1.5 ≤ WSI ≤ -1.0
WSL5: WSI ≤ -1.5

Year	**1996-2005**					**2006-2015**					
Regions	WSL1	WSL2	WSL3	WSL4	WSL5	WSL1	WSL2	WSL3	WSL4	WSL5	Total
Central	0	2.444	0.043	0	0	0	2.330	0.157	0	0	2.487
ENC	0	0.492	0.244	0.065	0	0.136	0.504	0.162	0	0	0.802
NE	0	0.662	0.932	0	0	0.002	1.296	0.295	0	0	1.594
NW	0	0.138	0	0	0	0	0.122	0.016	0	0	0.138
SE	0.401	1.719	0.297	0	0	0.106	1.900	0.410	0	0	2.416
South	0	2.053	0.085	0	0	0.337	1.696	0.105	0	0	2.138
SW	0.074	0.653	0.000	0	0	0	0.728	0	0	0	0.728
West	0.011	0.194	0	0.146	0.043	0.004	0.350	0.038	0.005	0	0.397
WNC	0	0.355	0.002	0	0	0	0.373	0	0	0	0.373
Total	0.486	8.710	1.603	0.211	0.043	0.584	9.299	1.184	0.005	0.000	11.073
% Total	4.39%	78.80%	14.51%	1.91%	0.39%	5.28%	83.98%	10.70%	0.05%	0.00%	100.00%

Year	**2016-2025**					**2026-2035**					
Regions	WSL1	WSL2	WSL3	WSL4	WSL5	WSL1	WSL2	WSL3	WSL4	WSL5	Total
Central	0.122	0.067	2.298	0	0	0	0	2.357	0.130	0	2.487
ENC	0	0.091	0.711	0	0	0	0	0.727	0.075	0	0.802
NE	0.040	0.238	1.317	0	0	0	0	1.272	0.322	0	1.594
NW	0	0	0.138	0	0	0.043	0.010	0.068	0.016	0	0.138
SE	0	0.266	1.613	0.537	0	0	0	2.162	0.255	0	2.416
South	0	0.056	2.045	0.037	0	0.006	0.014	0.694	1.424	0	2.138
SW	0	0.039	0.689	0	0	0.002	0.098	0.298	0.285	0.045	0.728
West	0	0	0.397	0	0	0	0.091	0.118	0.188	0	0.397
WNC	0	0	0.373	0	0	0	0	0.011	0.332	0.030	0.373
Total	0.161	0.758	9.580	0.574	0.000	0.051	0.214	7.706	3.026	0.075	11.073
% Total	1.46%	6.84%	86.52%	5.18%	0.00%	0.46%	1.93%	69.59%	27.33%	0.68%	100.00%

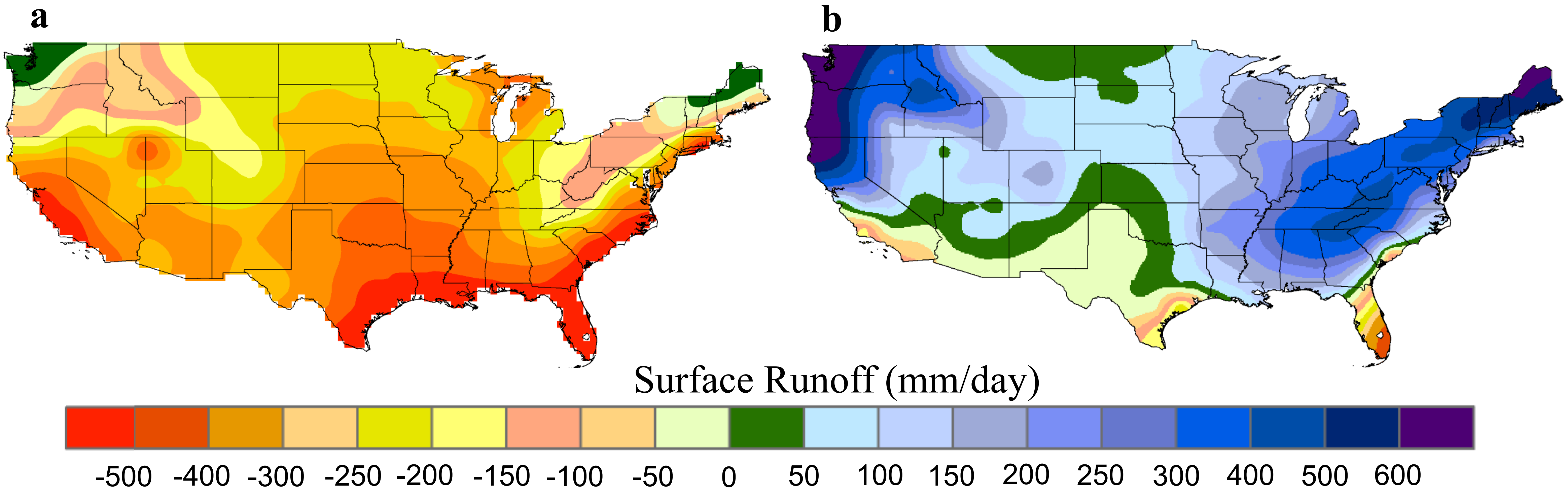
a
b
Surface Runoff (mm/day)
-500
-400
-300
-250
-200
-150
-100
-50
0
50
100
150
200
250
300
400
500
600

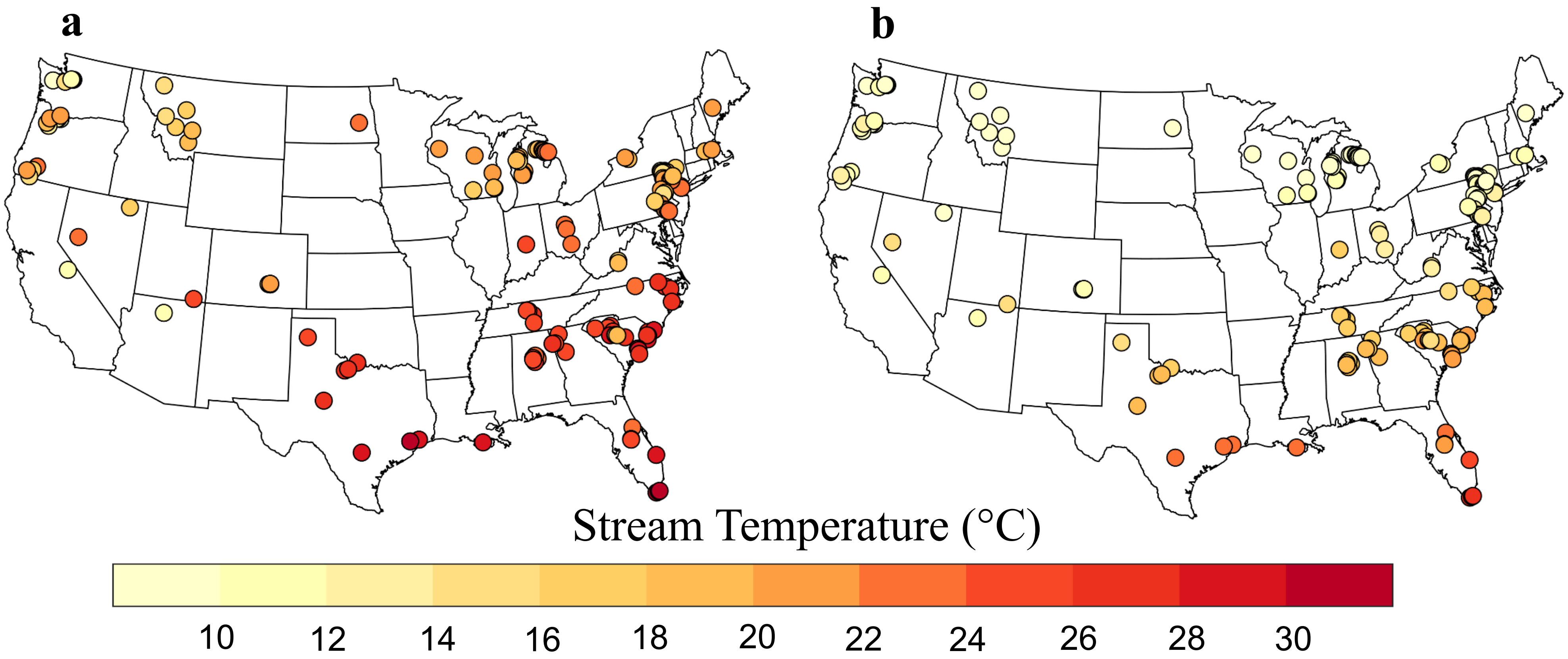
a
b
Stream Temperature (°C)
10
12
14
16
18
20
22
24
26
28
30

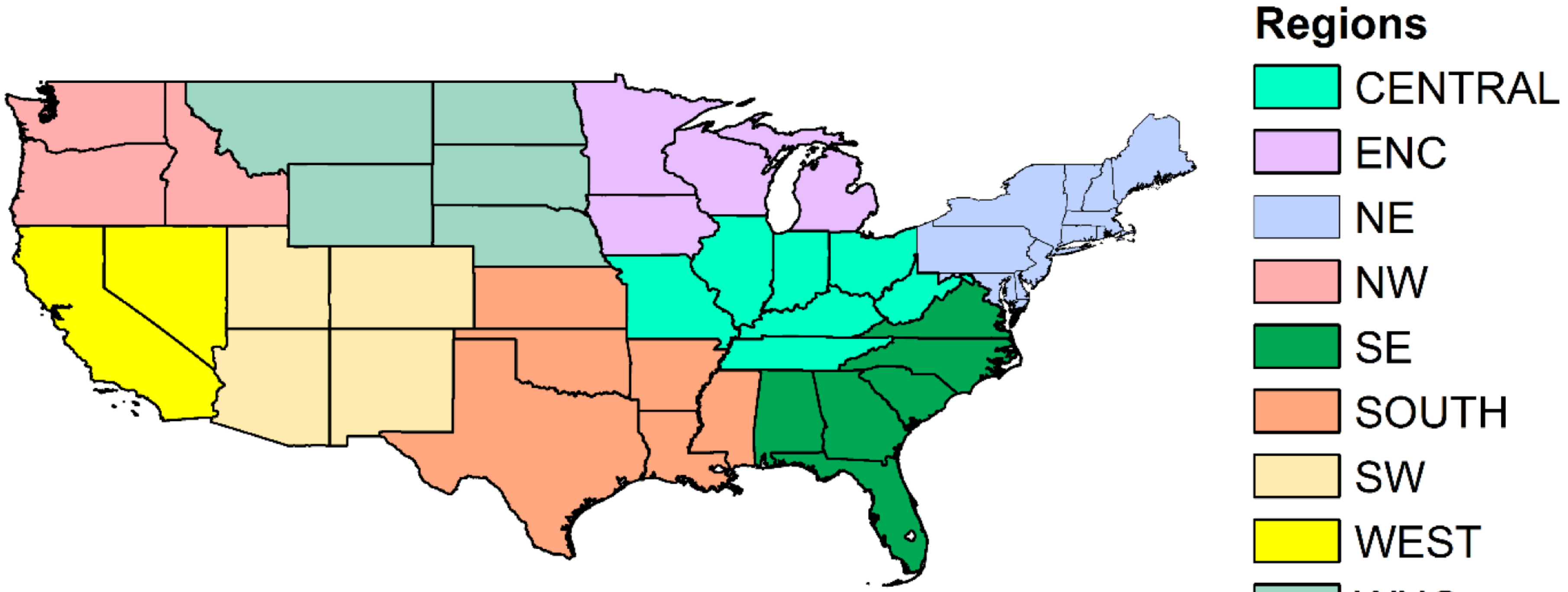
Regions
CENTRAL
ENC
NE
NW
SE
SOUTH
SW
WEST
WNC

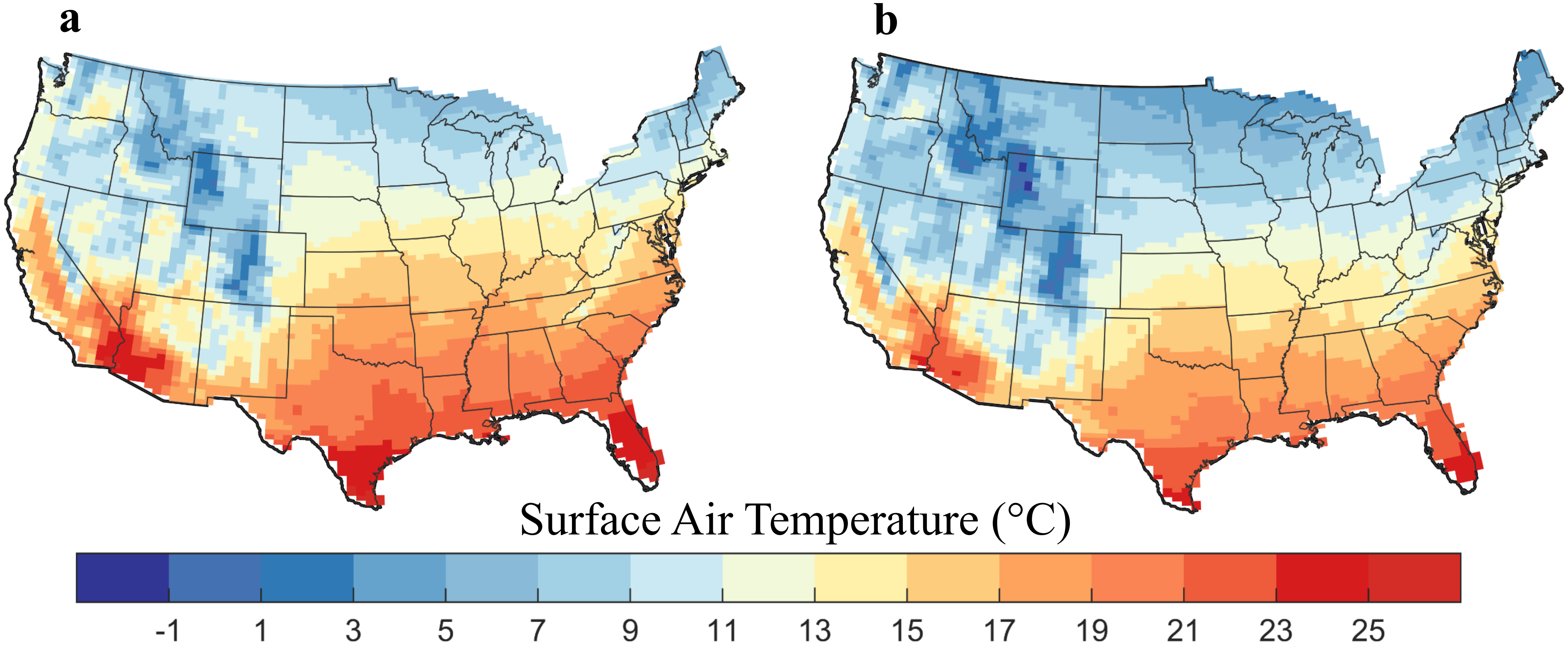

a
b
Surface Air Temperature (°C)
-1
1
3
5
7
9
11
13
15
17
19
21
23
25

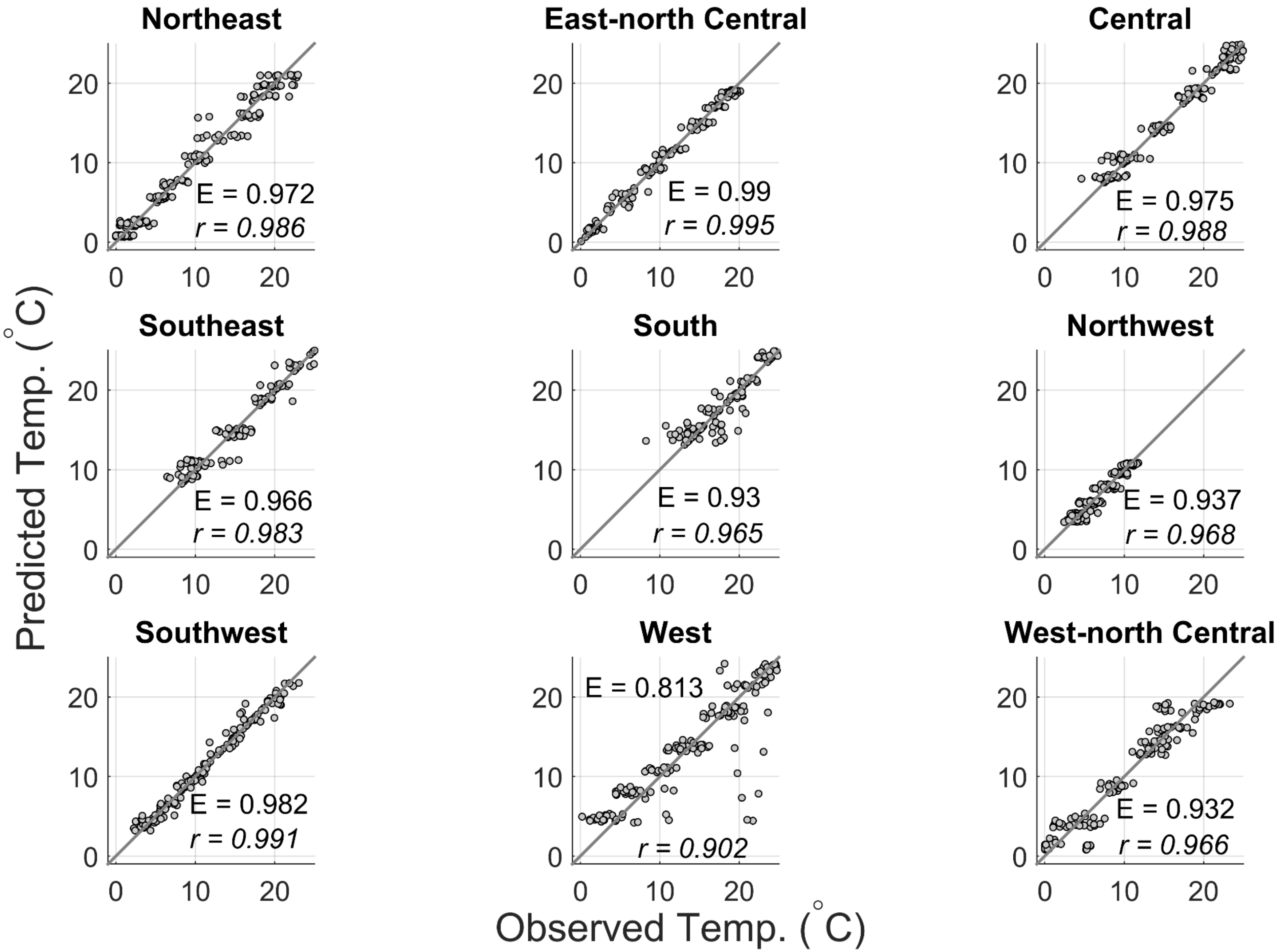

Northeast
E = 0.972
r = 0.986
East-north Central
E = 0.99
r = 0.995
Central
E = 0.975
r = 0.988
Southeast
E = 0.966
r = 0.983
South
E = 0.93
r = 0.965
Northwest
E = 0.937
r = 0.968
Southwest
E = 0.982
r = 0.991
West
E = 0.813
r = 0.902
West-north Central
E = 0.932
r = 0.966
Predicted Temp. (°C)
Observed Temp. (°C)

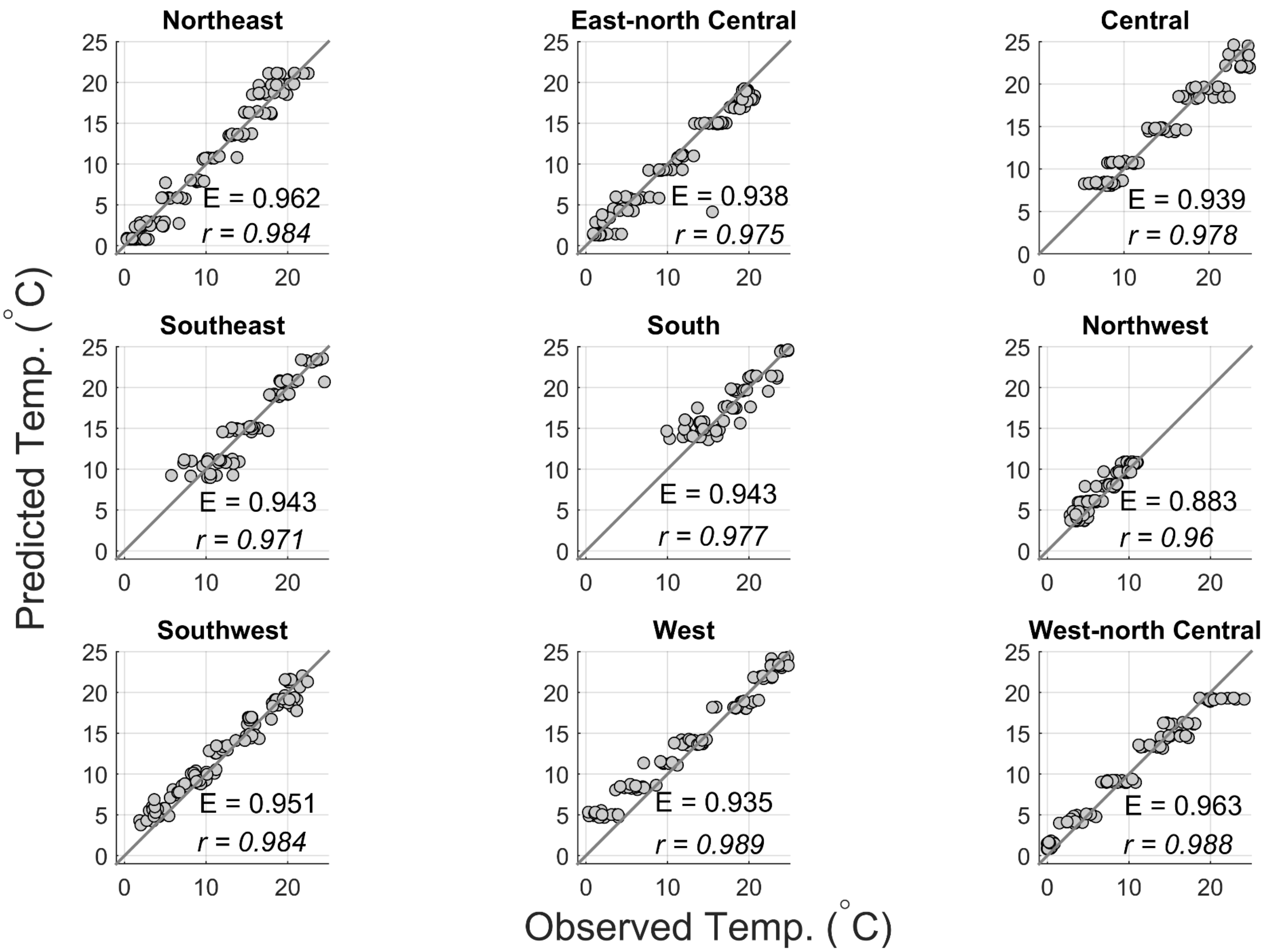

Northeast
E = 0.962
r = 0.984
East-north Central
E = 0.938
r = 0.975
Central
E = 0.939
r = 0.978
Southeast
E = 0.943
r = 0.971
South
E = 0.943
r = 0.977
Northwest
E = 0.883
r = 0.96
Southwest
E = 0.951
r = 0.984
West
E = 0.935
r = 0.989
West-north Central
E = 0.963
r = 0.988
Predicted Temp. (°C)
Observed Temp. (°C)
